# From neuromodulation to bone homeostasis: therapeutic targets of nerve growth factor in skeletal diseases

**DOI:** 10.3389/fphar.2025.1614542

**Published:** 2025-08-11

**Authors:** Kaixuan Chen, Longjun Chen, Yizhong Ma, Siqi Chen, Jinze Liu, Hangyu Zhou, Yuzhu Chen, Guanyi Liu

**Affiliations:** ^1^ Health Science Center, Ningbo University, Ningbo, China; ^2^ College of Medicine, Universitas Prima Indonesia, Medan, North Sumatra, Indonesia; ^3^ College of Chinese Medicine Materials, Jilin Agricultural University, Changchun, Jilin Province, China; ^4^ Department of Oncology, Beijing Chest Hospital, Capital Medical University, Beijing, China; ^5^ Department of Spine Surgery, Ningbo No.6 Hospital, Ningbo, Zhejiang Province, China

**Keywords:** nerve growth factor, bone homeostasis, bone formation, bone resorption, skeletal diseases

## Abstract

The skeletal system is an important support structure in the human body, and its homeostatic state is highly relevant to the development of a wide range of orthopaedic diseases. The search for key regulatory factors associated with skeletal development is essential for exploring potential therapeutic targets for bone diseases. Nerve Growth Factor (NGF), the first neurotrophic factor to be discovered, plays an important role in regulating immune cell function, influencing angiogenesis and participating in the physiological and pathological processes of bone homeostasis. Here, we mainly review the biological functions of NGF in the skeletal system and its molecular mechanisms, analyse the pathophysiological roles of the NGF signaling pathway in skeletal diseases such as osteoporosis, osteoarthritis, and fracture healing, and summarize the progress and challenges of the current clinical research on therapeutic strategies targeting NGF. In addition, we provide an overview of NGF and highlight the role of NGF in the regulation of bone formation and bone resorption. Therefore, by reviewing the literature related to NGF and bone diseases, this paper summarises the specific regulatory mechanisms of NGF in various bone diseases, which provides new perspectives and intervention targets for the treatment of skeletal diseases, especially in the field of diseases in which the effects of traditional treatments are limited. The therapeutic strategies targeting neurotrophic factors show broad prospects for clinical application.

## 1 Introduction

Nerve Growth Factor (NGF) as a neurotrophic factor (De Nardo et al., 2022), it is an important member of the Neurotrophin family (Amagai et al., 2016), which exhibits a typical characteristic folding pattern in its molecular structure. The family consists of four evolutionarily conserved secreted proteins, including NGF, Brain-Derived Neurotrophic Factor (BDNF), Neurotrophin-3 (NT-3) and Neurotrophin-4/5 (NT-4//5) (Aragona et al., 2022). These molecules all appear as homodimers in their three-dimensional structure, with each monomer containing approximately 120 amino acid residues that form a unique “neurotrophin fold” structural domain through three conserved disulfide bonds. The understanding of the functional diversity of NGF has been significantly deepened with the development of molecular biology research. Existing studies have shown that the biological effects of NGF have gone beyond its classical neurotrophic role and are widely involved in physiological processes such as immune regulation, angiogenesis and metabolic homeostasis. It is noteworthy that several studies in recent years have demonstrated that NGF plays an indispensable regulatory role in the development, homeostasis maintenance and pathological processes of the skeletal system. As a highly innervated organ, the development, metabolism and repair of bone tissue interact closely with the nervous system (Oo and Hunter, 2021), and NGF is one of the key molecules in mediating.

**FIGURE 1:**
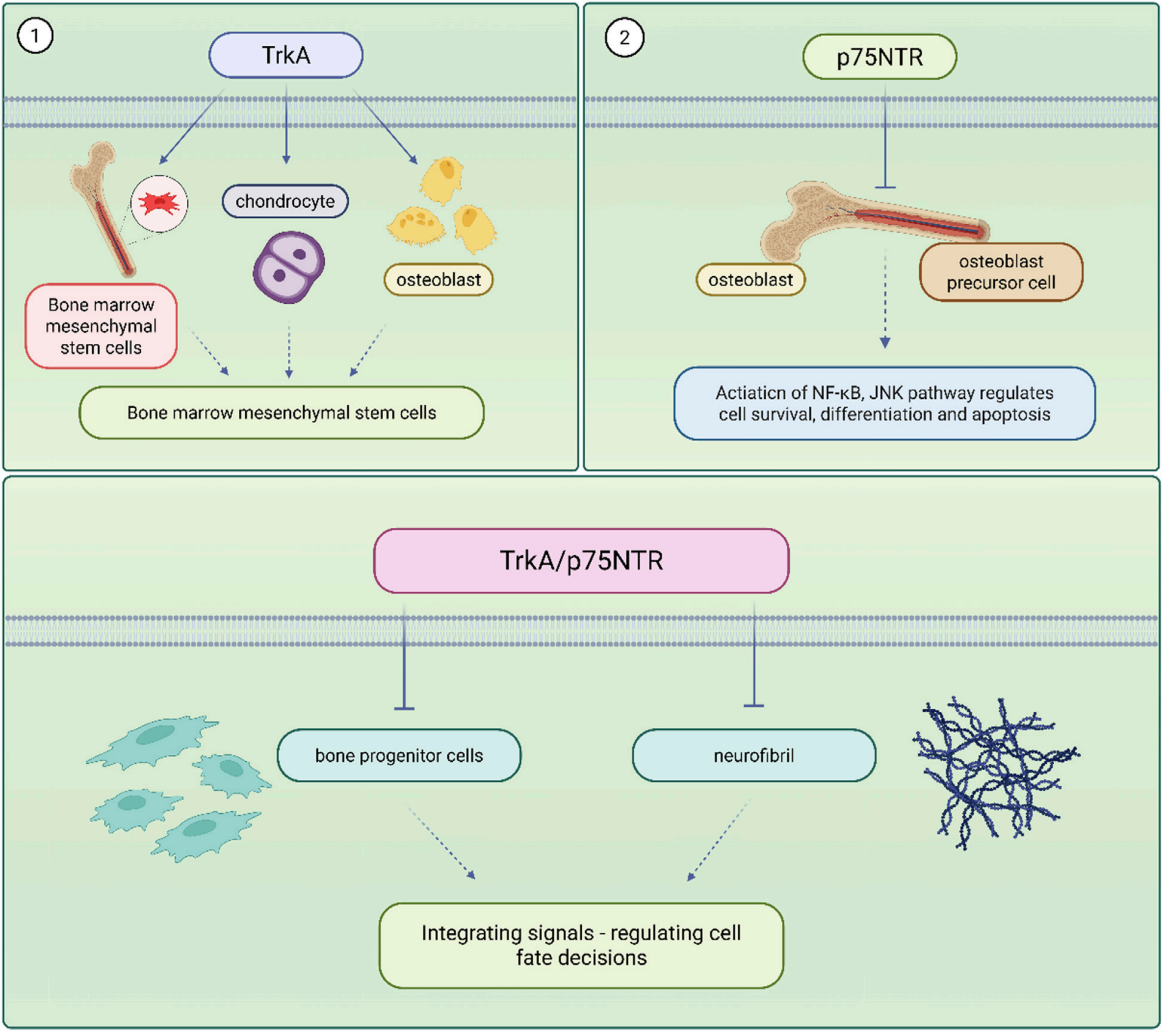
NGF receptor expression and function in skeletal cells.

Bone homeostasis refers to the dynamic equilibrium of bone formation, maintenance and resorption that ensures the strength and integrity of the skeleton and maintains a precise balance between osteoblast-mediated bone formation and osteoclast-mediated bone resorption (Mödinger et al., 2018). When this balance is disturbed, it can lead to pathological conditions such as osteoporosis and delayed fracture healing. Traditional treatments such as bisphosphonate (Giannasi et al., 2019), estrogen (Yang Y. et al., 2022) have been effective but suffer from high side effects and limited efficacy. Therefore, exploring new therapeutic targets has become an important direction in the study of skeletal diseases. There exists a complex and sophisticated two-way communication network between the skeletal system and the nervous system, and this relationship constitutes the theoretical basis of the ‘neural-skeletal axis’. Traditionally, the skeleton has been regarded as a mere support structure and mineral reservoir of the organism, but modern research has revealed that the skeleton is a highly dynamic and multi-regulated organ in which the nervous system is involved in the maintenance and repair of skeletal homeostasis through a variety of pathways. Nerve fibres are widely distributed in the periosteum, bone marrow and trabeculae, and directly or indirectly regulate the balance between bone formation and bone resorption through the release of neurotransmitters and NGF. The expression and function of NGF receptors in skeletal cells are shown in Figure 1.

It has been shown that NGF not only affects bone metabolism through innervation (Shi and Chen, 2024), but also acts directly on osteoblasts (Aso et al., 2024), to regulate their proliferation, differentiation and function. NGF signalling and its receptors have been shown to reduce the disease burden of painful musculoskeletal skeleton (Oo and Hunter, 2021), to target and regulate obesity and type 2 diabetes-induced skeletal muscle atrophy (Jun et al., 2024), and to promote vascular neovascularisation and skeletal muscle fibre remodelling in a mouse model of hindlimb ischaemia (Diao et al., 2022). These findings provide new perspectives for understanding the pathogenesis of skeletal diseases and a theoretical basis for developing novel therapeutic strategies.

**FIGURE 2:**
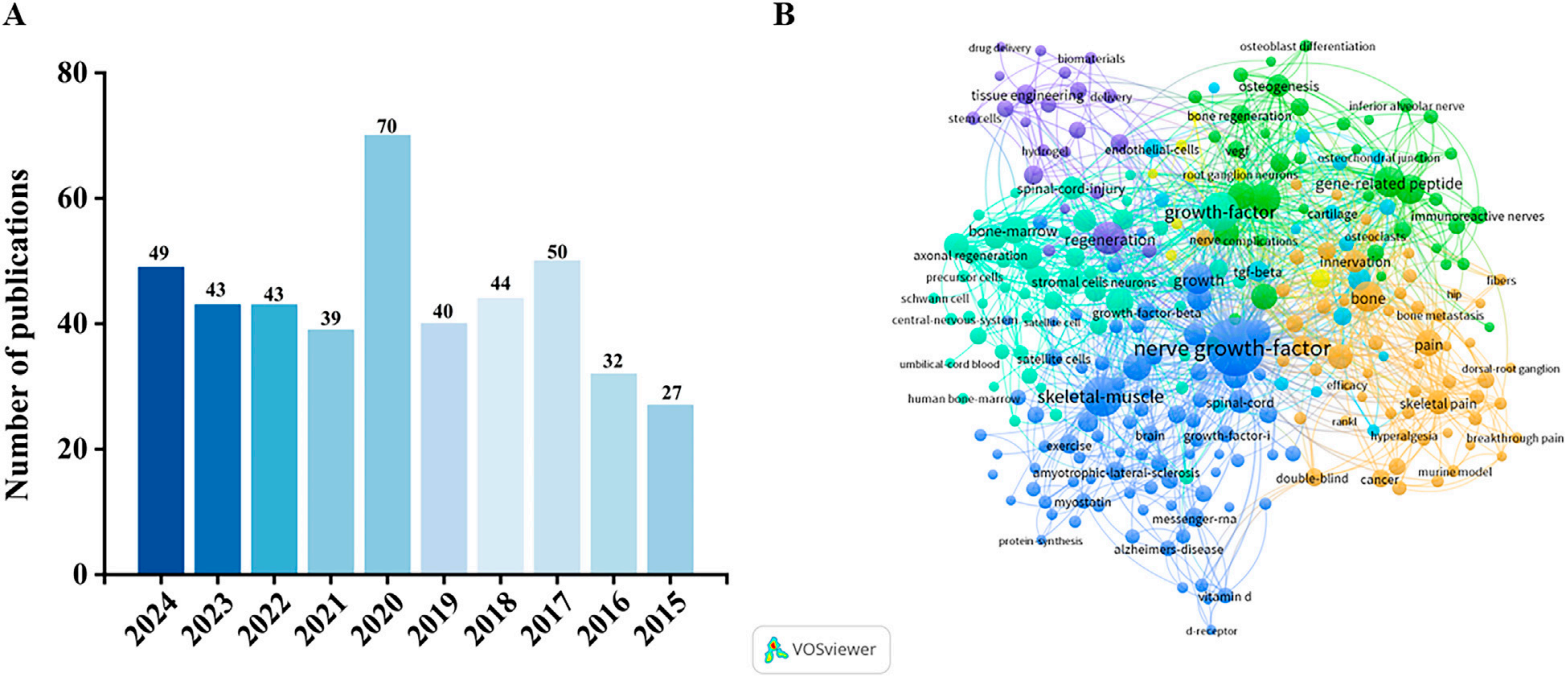
**(A)** Number of NGF-related publications in the Web of Science core collection in the past decade. **(B)** Keyword co-occurrence map based on bibliographic data created through VOSviewer 1.6.20 software (NGF, 2015–2024).

It is worth noting that the number of mentions of NGF has been increasing annually over the last decade, increasing to 70 in 2020, and then decreasing with mentions in the following years, but still remaining a hot topic (Figure 2A). Figure 2B shows the co-occurrence of keywords related to NGF. Among them, there are many keywords related to NGF, such as Bone homeostasis, Bone formation, Bone resorption, Skeletal diseases and so on. It can be seen that researchers are exploring more and more about NGF, and the functional mechanism of NGF regulation to the therapeutic target of bone homeostasis is getting more and more attention. The aim of this review is to systematically sort out the multiple functions of NGF from neuromodulation to maintenance of bone homeostasis, to deeply analyse its therapeutic potential in skeletal diseases, and to provide directions for future research by integrating basic research and clinical evidence to promote the innovative application of NGF in the treatment of skeletal diseases.

## 2 Methods

**FIGURE 3:**
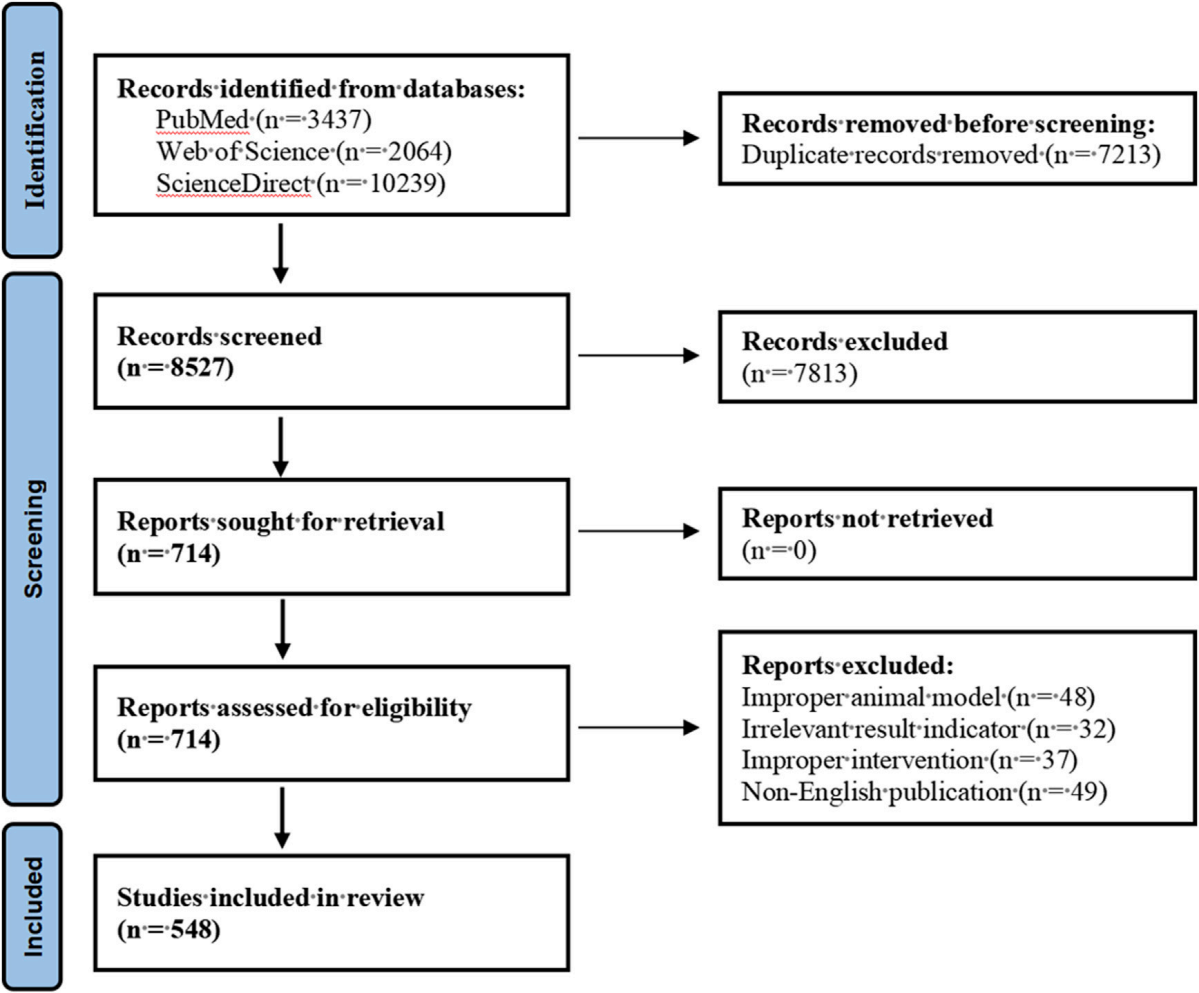
PRISMA flowchart of the literature screening.

In this study, we searched PubMed, Web of Science and ScienceDirect databases to obtain relevant literature resources. The search strategy was based on the combined keyword method, with Nerve growth factor, Bone homeostasis, Bone formation, Bone resorption and Skeletal diseases as the core subject terms to construct the search formula. We selected publications including animal experiments on Nerve growth factor and Bone homeostasis; research results on Bone homeostasis or Bone formation and Nerve growth factor; and analytical results on Nerve growth factor and Skeletal Diseases analysis results. Pharmaceutical preparations, non-English language and duplicate publications were excluded. This review strictly followed the systematic evaluation specifications of the PRISMA guidelines (Page et al., 2021), and the specific literature screening process is detailed in the visualisation flowchart shown in Figure 3.

## 3 Nerve growth factor overview

NGF is the earliest discovered and most thoroughly studied neurotrophic factor with dual biological functions of neuronal trophism and synapse growth, and it has an important regulatory role in the development, differentiation, growth, regeneration, and expression of functional properties of central and peripheral neurons.

### 3.1 Molecular structure and receptor system of nerve growth factor

NGF, BDNF, NT, NT-4 and NT-5 are collectively referred to as the NGF family due to their highly similar amino acid sequences, i.e., high degree of homology in gene structure. The molecular structure of NGF contains α, β, and γ subunits, with the active region being the β subunit, which is a homodimer consisting of the same subunits of two 118 amino acids bonded by non-covalent bonds, with three sets of disulfide bonds within each monomer. There are 3 groups of disulfide bonds in the monomer. The human nerve growth factor family members NGF, BDNF, NT-3 and NT-4. These protein sequences share about 50% homology (Aragona et al., 2022), and this high degree of conservation may be closely related to their functional specificity and receptor selectivity. In terms of genomic localisation, the human NGF gene is localised in the 1p13.1 region of the short arm of chromosome 1. In terms of expression regulation, NGF is subject to multiple levels of regulation, including promoter activity, enhancer regulation, transcription factor binding, and epigenetic modification, among other mechanisms. Notably, the expression of NGF shows significant tissue specificity, mainly distributed in neurons, immune cells and various glands and other tissues.

Mature NGF (β-NGF) is a homodimer consisting of two identical subunits, each containing 112 amino acid residues (Zhou et al., 2021), and a molecular weight of 13.5 kDa (Lim et al., 2007). The precursor protein proNGF is encoded by NGF and is initially translated into a precursor protein containing about 240 amino acids (preproNGF), which is cleaved by a signal peptide to form proNGF, an N-terminal pre-structural domain and a C-terminal mature NGF structural domain (Feng et al., 2010). In the Golgi or secretory vesicles, the precursor convertase cleaves proNGF and releases β-NGF proNGF secreted outside the cell can be further processed by extracellular proteases. If processing is incomplete, proNGF may exist in full-length form. In the nervous system proNGF is more stable, whereas in peripheral tissues it is susceptible to rapid processing into NGF, and proNGF accumulation in pathological states may result from an imbalance in protease activity (Fahnestock et al., 2004).

**FIGURE 4:**
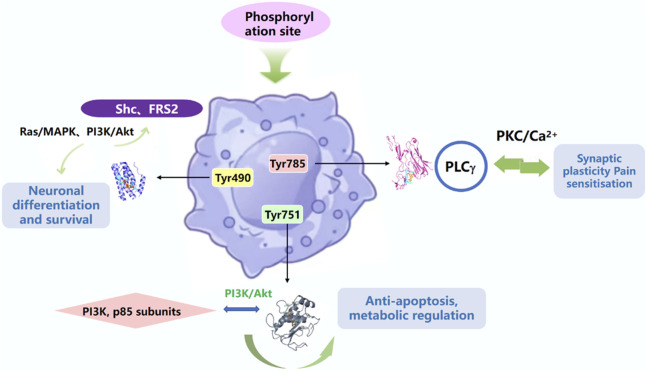
TrkA activation is dependent on intracellular specific tyrosine residues phosphorylating sites of downstream signalling proteins.

NGF acts by binding to specific cell surface receptors, including the high-affinity receptor TrkA and the low-affinity receptor p75NTR, which together regulate the diverse biological effects of NGF. TrkA activation involves ligand-induced dimerisation, autophosphorylation, and initiation of downstream signalling cascades, as well as the synergism of multiple pathways (MAPK, PI3K, PLCγ), which are dynamically balanced and ultimately regulate neuronal survival, differentiation, synaptic plasticity and other biological processes, dynamic homeostasis, and ultimately regulates biological processes such as neuronal survival, differentiation, and synaptic plasticity (Figure 4).

p75NTR is a member of the tumour necrosis factor receptor superfamily and regulates cell survival and apoptosis in the nervous system with its unique dual role (Zhou et al., 2019). The dual regulatory mechanism of p75NTR promotes survival signalling, amplifies the PI3K/Akt and Ras/MAPK pathways by enhancing the ligand-binding affinity of the Trk receptor, and p75NTR-TRAF6 signalling promotes NF-κB nuclear translocation and upregulates anti-apoptotic genes to activate the NF-κB pathway. NGF exerts its physiological effects by binding two classes of cell surface receptors high affinity TrkA receptors and low affinity p75NTR. A 2020 report suggests that NGF plays an important role in the plasticity and survival of cholinergic neurons in the basal forebrain associated with cognitive function. TrkA/p75NTR is an important factor in neuronal apoptosis. TrkA/p75NTR and PI3K/AKT pathways were shown to be involved in the regulation of intermittent hyperglycaemia (IHG)-induced neuronal apoptosis and the neuroprotective effects of alpha-lipoic acid (ALA) under IHG (Yan et al., 2020). Both p75NTR and TrkA play important roles in the nervous and skeletal systems. The development of targeted drugs with higher specificity, avoiding effects on non-target tissues, is key to achieving NGF therapy. By selectively modulating the activity of TrkA and p75NTR, side effects can be reduced and therapeutic efficacy improved.

### 3.2 Physiological functions of nerve growth factor

#### 3.2.1 Core functions in the nervous system

Precise regulation of neuronal survival and apoptosis, NGF-TrkA activates PI3K-Akt, Ras-MAPK, and maintains the survival of sympathetic and dorsal root ganglion (DRG) sensory neurons (Kigami et al., 2021). Activation of the PI3K-Akt pathway inhibits apoptosis, while the Ras-MAPK pathway promotes cell proliferation and differentiation. The synergy of these signalling pathways ensures stable neuronal survival during development and maturation. Under normal physiological conditions, the presence of NGF is essential for maintaining the survival of sympathetic and DRG sensory neurons. However, when NGF is deprived or its precursor form proNGF accumulates, p75NTR forms a complex with sortilin that activates the JNK-c-Jun pathway, which in turn induces mitochondrial apoptosis via Bim and PUMA. This mechanism is particularly important in neurodegenerative diseases because it determines neuronal survival or death. Axon-directed chemical gradients decode NGF into concentration gradients in target tissues skin or vasculature, etc., and growth cones dynamically regulate the cytoskeleton through local TrkA internalisation with Rho GTPase. NGF and its receptors TrkA and p75NTR play key roles in several physiological and pathological processes in the nervous system. From neuronal survival and apoptosis to axon guidance and regeneration to synaptic plasticity and learning memory, NGF regulates these complex processes through the activation of different signalling pathways. In the ventral spinal cord, Semaphorin3A induced growth cone collapse via the p75NTR-NRP1 complex. After nerve injury, regenerating axons can re-respond to NGF gradients, but proNGF-p75NTR signalling in scar tissue may hinder regeneration. Dynamic regulation of synaptic plasticity, basal forebrain NGF-TrkA signalling maintains cortical cholinergic projections and modulates learning memory. NGF enhances LTP via the TrkA-PLCγ-CaMKIV pathway, and proNGF-p75NTR inhibits LTP retrograde transport of NGF released from target tissues into neuronal cytosol regulates synaptic protein expression.

In addition, Yiangou et al. showed accumulation of immunoreactivity in nerve fibres distal to the rat sciatic nerve ligature in both normal human and rat skin tissue sections immunostained for rhNGF and antibodies to preNGF immunostained for basal keratin-forming cells, suggesting that prepro-NGF may be preferentially uptaken and transported by p75 (Yiangou et al., 2002). The monocyte or macrophage-driven NGF-TrkA pathway was found to be a novel analgesic target for adult survivors of childhood cancer in a rat model of cisplatin-induced survival pain (Da Vitoria Lobo et al., 2024).

#### 3.2.2 Pleiotropic effects in the non-neurological system

Recent studies have revealed that NGF and its receptors TrkA and p75NTR are widely expressed in non-neural tissues, demonstrating pleiotropic effects. NGF is secreted by mast cells, macrophages, etc. It can activate basophils to release histamine, promote B-cell proliferation and T-cell differentiation, and participate in Th2-type immune responses (Minnone et al., 2017). However, this immunomodulation is early in the course of infection, where NGF promotes tissue repair by enhancing the Th2 response. In chronic inflammation, its sustained release exacerbates the fibrotic process. The NGF system appears to play a role in the reproductive physiology of this grey squirrel population by participating in the regulation of the ovarian cycle mainly through local regulation of NGF/NGFR (Maranesi et al., 2020).

#### 3.2.3 Role in the inflammatory response

NGF exhibits unique bidirectional regulation in the inflammatory response, directly binding TrkA/p75NTR (Moyano et al., 2021). Promotes mast cell degranulation and release of histamine, TNF-α and proteases. Activates the NF-κB and MAPK pathways through TrkA, promotes M1-type polarisation, and increases the secretion of IL-6, IL-1β and NO to exert pro-inflammatory effects (Ruggeri et al., 2016). NGF activates Foxp3 expression via p75NTR to promote Treg proliferation and inhibit excessive immune response; during the tissue repair phase, NGF induces Arg-1 and IL-10 expression via the PI3K-Akt pathway to promote M2-type transformation (Sainath and Gallo, 2021). Notably, sustained activation of p75NTR by p75NTR-mediated immunosuppression in a lung fibrosis model promotes cross-talk of the TGF-β1-Smad2/3 pathway, resulting in aberrant fibroblast activation (Peng et al., 2019). NGF enhances tight junction protein expression in intestinal epithelial cells via TrkA and attenuates DSS-induced colitis.

#### 3.2.4 Regulation of vascular endothelial cell proliferation and angiogenesis

NGF is involved in the proliferation, migration and angiogenesis of vascular endothelial cells (ECs) through direct and indirect mechanisms. It plays a key role in both physiological angiogenesis and pathological vascular proliferation. NGF-TrkA inhibits endothelial cell apoptosis through Akt phosphorylation. NGF induces IL-10 and TGF-β secretion from M2-type macrophages, which promotes vascular maturation, and sympathetic-dependent fibres through norepinephrine regulates vascular tone (Lin et al., 2024; Yu et al., 2019). Glioblastoma, prostate cancer highly express NGF, which promotes angiogenesis through autocrine/paracrine secretion (Baspinar et al., 2017; Shimomura et al., 2021). While anti-NGF antibodies inhibit vascular proliferation, they simultaneously block innervation-mediated vascular maturation signalling (Troullinaki et al., 2019).

## 4 Regulation of bone formation by NGF

### 4.1 Skeletal system

NGF played a complex regulatory role in skeletal development, homeostasis maintenance and disease processes (Li et al., 2019). The skeletal system being a highly innervated and dynamically remodelled tissue, NGF is involved in bone formation, bone resorption, pain perception and injury repair through its receptors TrkA and p75NTR. The bone remodelling process is mainly regulated by osteoclasts and osteoblasts, and the dynamic balance of their functions is essential for maintaining bone homeostasis (Moradinasab et al., 2021). Neurotrophic factors such as NGF have been shown to have significant osteogenic stimulatory effects, emphasising the intricate interplay between neurotrophism and bone healing (Li et al., 2025). Sekiguchi et al. found a relative increase in NGF in healing tissues after fracture in a mouse femur fracture model, suggesting that the organism has an increased need for neurotrophic and skeletal remodelling after fracture. The intricate linkages between these systems and factors highlight the complex role of NGF in skeletal remodelling (Sekiguchi et al., 2022).

### 4.2 NGF promotes bone formation through proliferation and differentiation of bone marrow mesenchymal stem cells

NGF significantly enhances the osteogenic efficacy of bone marrow mesenchymal stem cells (BMSCs) through a multi-targeted regulatory mechanism and plays a key regulatory role in the bone formation process (Yang et al., 2019). In the process of fracture repair, NGF-regulated BMSCs accurately coordinate the healing phase transition of ‘inflammation-chondrogenesis-osteogenesis-remodelling’ through specific differentiation into osteoblasts and chondrocytes, and at the same time secrete a variety of extracellular matrix proteins (e.g., collagen type I, bridging proteins) to promote the deposition and mineralisation of the bone matrix. NGF is a key molecule connecting cell aggregation in the early stages of bone formation and tissue reconstruction in the later stages of bone formation. BMP-2 is widely recognised as the most osteoinductive growth factor, which is effective in the repair of bone defects and fracture healing. (Ye and Ma, 2019). Experiments have confirmed that BMP-2 can effectively promote the differentiation of MSCs to osteoblasts, enhance ALP activity and osteocalcin (OCN) expression, and significantly increase the amount of new bone formation and the quality of bone healing (Li et al., 2022). In addition, NGF indirectly promotes the osteogenic potential of BMSCs by modulating the bone marrow microenvironment (Zhao H. et al., 2025). On the one hand, NGF stimulates BMSCs to secrete a variety of osteogenesis-related cytokines (e.g., BMP-2, VEGF), forming an autocrine/paracrine positive feedback regulatory loop (Yang X. et al., 2022). BMP-2 is widespread in the skeletal system, especially in osteoblasts and osteocytes. It plays a key role in bone formation, promoting bone tissue production by inducing intramembranous ossification and endochondral ossification (Halloran et al., 2020). On the other hand, NGF inhibits the expression of the adipose differentiation-associated transcription factor PPARγ and reduces the differentiation of BMSCs to adipocytes, thereby directing more stem cells to the osteogenic lineage (Suo et al., 2022). Animal experiments have confirmed that local application of NGF can increase the recruitment of BMSCs by 3-fold and new bone formation by more than 50% in a bone defect model (Rivera et al., 2020). By promoting osteogenic differentiation of MSCs, NGF plays an important role in promoting bone formation.

### 4.3 NGF regulates bone formation by promoting osteoclast differentiation and maturation

Osteoclasts, as the main functional cells for bone resorption, are specialised multinucleated giant cells formed by the fusion of precursor cells of the monocyte/macrophage lineage (Huang et al., 2024; Konno et al., 2024). During osteoclast differentiation and maturation, osteoblasts are able to secrete active factors associated with coupling. These active factors enable osteoclasts to interact with osteoblast precursor cells, promoting osteoblast differentiation and mineralisation, which is critical for maintaining the balance between bone resorption and bone formation during bone remodelling (Kim et al., 2020). It has been found that NGF and its high affinity receptor TrkA are co-expressed in mature osteoblasts, suggesting that NGF may be involved in the regulation of osteoblast function through an autocrine/paracrine mechanism (Delay et al., 2022). NGF stimulates the secretion of RANKL (factor essential for osteoclast differentiation) from osteoblasts and bone stromal cells, and may inhibit the expression of osteoprotegerin (OPG), thus indirectly promoting osteoclastogenesis, but this effect may be amplified under inflammatory conditions. NGF binds to TrkA and p75NTR receptors on the surface of osteoclast precursor cells, activates the NF-κB and MAPK signalling pathways, significantly upregulates the expression of RANKL, and promotes the differentiation of osteoclast precursor cells to mature osteoblasts, which exacerbates bone resorption in osteoporosis models. Meanwhile, NGF promotes the bone resorption function of mature osteoclasts by enhancing the activities of osteoclast-specific markers such as histone kinase K (CTSK) and anti-tartaric acid phosphatase (TRAP), which maintains the normal bone remodelling process (Walia et al., 2018). NGF plays a key role in the dynamic balance between bone formation and bone remodelling through mechanisms that precisely regulate osteoclast activity.

### 4.4 NGF regulates bone formation through nerve-vessel coupling

The skeleton is a highly innervated organ in which nerve fibres interact with various skeletal cells. Peripheral nerve endings release neurogenic factors and sense skeletal signals that mediate bone metabolism and skeletal pain (Sun et al., 2023). Bone is also one of the tissues with the richest vascular network in the body, and the vascular system is crucial for bone development, homeostasis and regeneration. During bone development, regeneration and remodelling, vascular neovascularisation not only provides oxygen and nutritional support to bone tissue, but also directly participates in osteogenic regulation through the secretion of various paracrine factors (e.g., PDGF, VEGF) (Liu et al., 2023). Innervation is a key initiator of the bone repair process, which precedes the bone regeneration process (Li et al., 2019). Studies have shown that the absence of the NGF signalling pathway leads to multiple pathological changes: abnormal sensory innervation, delayed neovascularisation, reduced numbers of bone progenitor cells and impaired skeletal development (Tomlinson et al., 2016). Bone regeneration is a multi-system process: the vascular system provides the necessary nutrients and oxygen supply, the immune system regulates the inflammatory response, and the nervous system coordinates this complex process through the secretion of neurotransmitters (Leroux et al., 2020). Clinical observations show that patients with congenital insensitivity to pain (CIP) are significantly more likely to develop skeletal complications such as fractures and bone infections (Zhang and Haga, 2014). Neurological abnormalities are also closely associated with a number of skeletal diseases, such as osteoporosis, which is often associated with abnormal sympathetic nerve activity, and osteoarthritis, in which the density of joint innervation is reduced by about 40% (Zhu et al., 2019). Li et al. revealed for the first time the regulation of neurovascular regeneration during fracture repair. First, *in vivo* microimaging confirmed that the reinnervation of sensory nerves to the bone scab significantly preceded the formation of neovascularisation; second, three-dimensional reconstruction analysis showed that there was a high degree of spatial correlation between the distribution trajectories of nerve fibres and blood vessels; what’s more, for the first time, the study confirmed that the peak of NGF expression at molecular level was exactly between the peak of nerve growth and the peak of vessel growth. Between the peak of nerve growth and the peak of vascular growth, suggesting that NGF may be a key molecular bridge connecting nerve regeneration and neovascularisation, providing direct evidence for understanding the neurovascular coupling mechanism of fracture healing (Li et al., 2019). Studies of NGF in the regulation of bone formation are shown in Table 1.

**TABLE 1:** NGF in the regulation of bone formation.

Research directions	Main findings	Experimental models/Methods	References
NGF expression in bone tissue	NGF is secreted by osteoblasts, osteoclasts and nerve fibres and plays a bidirectional regulatory role in bone metabolism	TrkAF592A mice and TrkAWT; X-Gal staining, transparency and confocal imaging, quantitative RT-PCR, histology	Tomlinson et al. (2017)
Effect of NGF on chondrocyte angiogenesis *in vitro*	NGF promotes FGF2 expression and increases TrkA-mediated angiogenesis via PI3K/Akt and ERK/MAPK signalling pathways in human chondrocytes *in vitro*	OA patient model; NGF and TrkA expression in cartilage detected by immunohistochemistry and WB, Safranin-O	Yu et al. (2019)
The role of NGF in fracture healing	Upregulation of NGF expression at fracture sites promotes nerve regeneration and blood vessel formation to accelerate healing	Patients with clavicle fracture combined with TBI; serum NGF levels by ELISA and immunohistochemistry	Zhang et al. (2018)
Impact of NGF on bone defect repair	NGF promotes BMSC osteogenesis and accelerates bone repair	Sprague-Dawley (SD) rat femoral condylar defect model; *in vitro* and *in vivo* micro CT, histological, WB and PCR analyses	Ji et al. (2024)
NGF in models of osteoporosis	NGF levels are reduced in a postmenopausal osteoporotic mouse model; NGF supplementation partially improves bone mineral density	Ovariectomy (OVX) Large osteoporotic bone defect model; RNA sequencing, TRAP staining	Zhao Y. et al. (2025)
Application of NGF in osseointegration of oral implants	NGF promotes differentiation of BMSCs to osteoblasts and neuronal cells	Animal implant experiments, histological analysis	Ye and Gong (2020)
Synergy between NGF and calcitonin gene-related peptides	CGRP and NGF work together to promote bone regeneration, and CGRP does so by increasing angiogenesis and osteoblast activity	Mandibular defect model with combined CGRP and NGF intervention	Sun et al. (2015)
The role of NGF in mechanical stress response	Mechanical loading (e.g., exercise) enhances osteogenic activity by upregulating NGF expression	C57BL/6J mice; histomorphological, qRT-PCR *in vitro* detection of Ngf, Wnt1 and Wnt7b expression in ATCC CRL-2593	Fioravanti et al. (2021)
Effects of NGF inhibitors on pain aspects of osteoarthritis	Effects of anti-NGF treatment (e.g., tanezumab) blockade on pain behaviour and joint structure in an experimental model of osteoarthritis	——	Miller et al. (2017)
NGF and inflammatory bone loss	NGF overexpression under inflammatory conditions exacerbates osteoclast activation and leads to bone erosion	Primary cultures of human OA chondrocytes, neonatal mouse articular chondrocytes, or cartilage explants were stimulated by increasing IL-1β, prostaglandin E2, mucin/nicotinamide phosphoribosyltransferase, or cyclic mechanical compression	Pecchi et al. (2014)
Potential therapeutic role of NGF in osteoarthritis (OA)	NGF promotes chondrocyte proliferation and matrix synthesis	Clinical studies and animal OA models	Shang et al. (2017)

## 5 Regulation of bone resorption by NGF

### 5.1 Direct action of NGF on osteoclasts

Osteoclasts are the main functional cells for bone resorption and have an important role in bone development, growth and injury repair. When mature osteoclasts adhere to the bone surface, osteoclasts are activated and begin bone resorption (Chen Z.-H. et al., 2023). Once the function of osteoclasts is abnormal, bone resorption will be disturbed, breaking the balance between bone formation and resorption. When osteoclasts function is in a hyperactive state, it will cause bone degenerative diseases such as osteoporosis, arthritis, etc. If osteoclasts are dysfunctional or in decline, they can cause bone resorption disorders, osteosclerosis, dense osteogenesis imperfecta, Paget’s disease of bone, and massive osteolysis (Xiang et al., 2024). By virtue of its unique molecular structure and properties, NGF can accurately bind to specific receptors on the surface of osteoclasts. Under the direct action of NGF, osteoclasts showed significant changes in cell morphology, functional activity and gene expression profiles. From the perspective of cell morphology, the pseudopod extension and cell polarity of osteoclasts will be adapted to the needs of bone resorption function (Chen Z.-H. et al., 2023). In terms of functional activity, osteoclasts have a regulated ability to degrade bone matrix, which directly affects the rate and extent of bone resorption by secreting a variety of acids and proteases, such as lactic acid, citric acid, CTSK, etc., which solubilise and break down the bone matrix (Vääräniemi et al., 2009). It has been shown that NGF presents differences in the regulation of osteoclasts under different pathophysiological states. In inflammatory environments, the release of inflammatory cytokines such as TNF-α and IL-6 affects the expression of NGF and its receptor on osteoclasts, as well as signalling, and TNF-α upregulates the expression of the NGF receptor on osteoclast precursor cells, which enhances the promotion of osteoclast differentiation, thereby exacerbating bone resorption (Yao et al., 2022). In addition, progress has been made in the study of the interaction between NGF and other bone regulators. For example, NGF has a synergistic or antagonistic relationship with members of the BMP family in regulating bone metabolism; BMP-2 promotes osteoblast differentiation and bone formation, whereas NGF regulates the BMP-2 signalling pathway to a certain extent, affecting the balance between osteoblasts and osteoclasts, and indirectly regulating bone resorption (Yu, 2000). NGF also directly promotes bone resorption by enhancing CTSK and V-ATPase proton pump activity and increasing the bone matrix degradation capacity of mature osteoclasts by up to 2-fold. NGF further optimises the bone resorption microenvironment by up-regulating osteoclast crease margin formation and MMP-9 secretion (McConnell et al., 2017).

### 5.2 Indirect regulation through neuroendocrine

NGF, as a core substance of neuromodulation, has a significant regulatory effect on neuroendocrine cells, inducing them to secrete various hormones and neuropeptides, which are then involved in the complex regulation of bone metabolism. These substances are then involved in the complex regulation of bone metabolism. NGF in the central nervous system indirectly alters peripheral bone metabolism by regulating the secretion of metabolism-related neuropeptides, such as leptin and NPY, which inhibit bone formation and promote bone resorption through Y1 receptors, and leptin, which inhibits bone formation through the sympathetic efferent pathway, thus forming a complex network of neuroendocrine-bone metabolism regulation (Liang et al., 2025). In addition, NGF regulates the hypothalamic-pituitary axis to influence bone metabolism. In the hypothalamic-pituitary-adrenal axis, NGF stimulates specific neurons in the hypothalamus to alter the secretion of corticotropin-releasing hormone (CRH), which in turn affects the pituitary’s secretion of adrenocorticotropic hormone (ACTH), which travels with the bloodstream to the adrenal cortex, where it regulates the synthesis and release of cortisol (Chen M. et al., 2023; Sheng et al., 2021). NGF became a key regulatory molecule in the process of bone resorption, and Table 2 shows the therapeutic targets of NGF in bone resorption.

**TABLE 2:** Therapeutic targets of nerve growth factor in bone resorption.

Target	Relevance	Potential applications	References
Inhibition of the NGF-TrkA signalling pathway	Blocking NGF binding to TrkA receptors reduces osteoclast activation	Osteoporosis, rheumatoid arthritis	Yamashiro et al. (2000)
Downregulation of the p75NTR signal	Inhibition of p75NTR-mediated osteoclast differentiation and bone resorption activity	Bone metastatic cancer, Paget’s disease	Douillard et al. (2016)
Adjustment of RANKL/OPG balance	NGF regulates bone resorption by affecting the ratio of RANKL and OPG	Postmenopausal osteoporosis	Stapledon et al. (2021)
Inhibition of the NF-κB pathway	NGF promotes osteoclastogenesis through activation of NF-κB and targeted inhibition reduces bone loss	Inflammatory bone destruction	Wei et al. (2020)
Modulation of TRPV1 channels	NGF enhances nociception and bone metabolism via TRPV1, and antagonising TRPV1 reduces bone resorption	Painful Bone Diseases	Obeidat et al. (2020)
Antibodies targeting NGF (e.g., Tanezumab)	Neutralisation of NGF activity, reduction of osteoclast activation and pain signalling	Chronic osteodynia, osteoporotic fracture	Xu et al. (2016)
Modulation of sympathetic signalling	NGF affects bone metabolism via sympathetic nerves; targeted intervention improves bone reconstruction	Neurogenic bone loss	Zhang Z. et al. (2023)
Inhibition of inflammatory factors	NGF promotes the release of inflammatory factors and indirectly activates osteoclasts, and anti-inflammatory treatment may synergise against bone resorption	Autoimmune bone disease	Aso et al. (2019)
Activation of the Wnt/β-catenin pathway	Antagonism of NGF inhibition of Wnt signalling promotes osteoblast differentiation to balance bone metabolism	Low bone formation osteoporosis	Rivera et al. (2020)
Regulation of autophagic processes	Reduction of NGF secretion	Bone tumor therapy	Zhang et al. (2025)

## 6 Therapeutic potential of nerve growth factor in skeletal diseases

### 6.1 Arthritis

**FIGURE 5:**
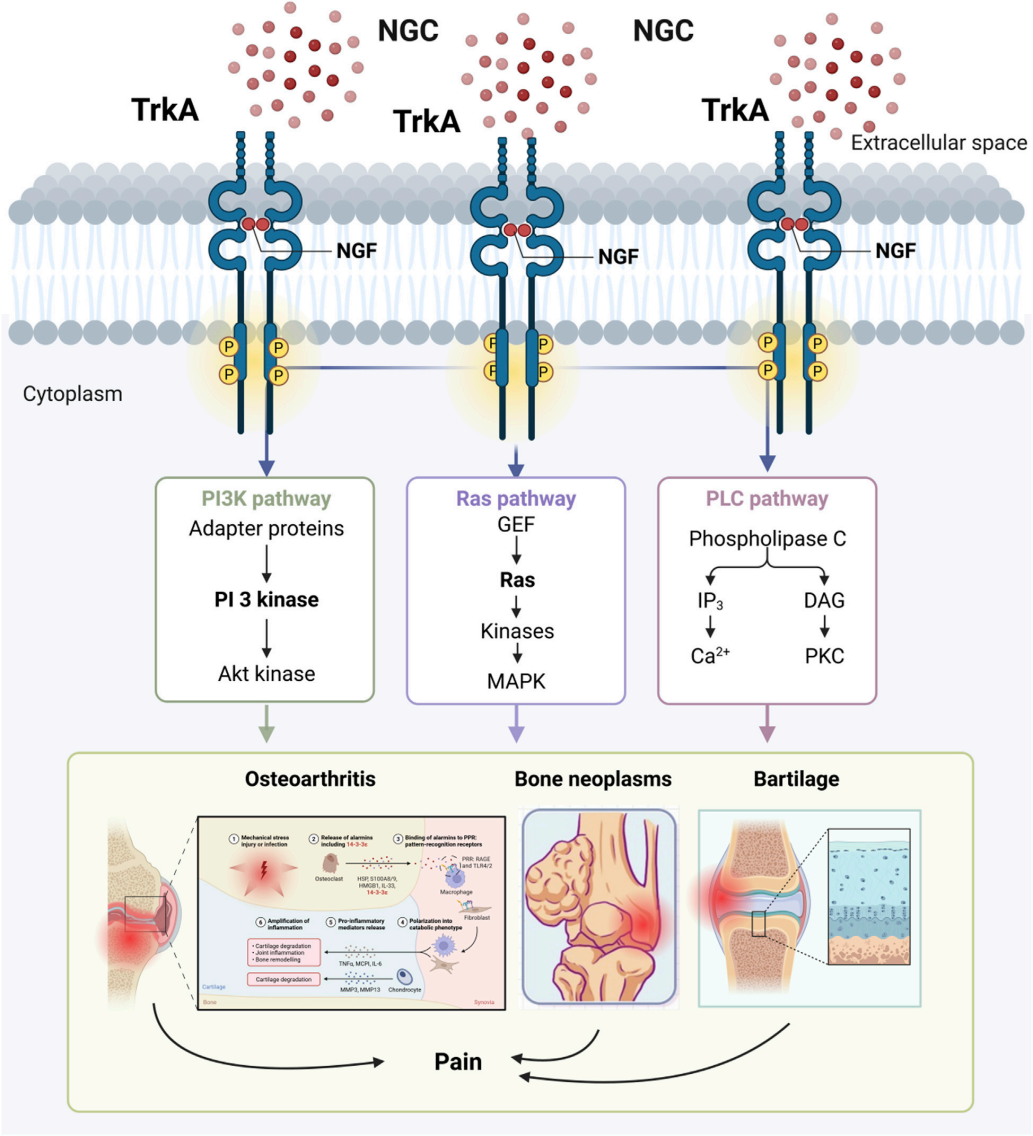
NGF affects the development of bone-related diseases through activation of the receptor TrkA.

Nerve growth factor not only participates in osteochondral pathological processes via neurovascularity, but also acts as an inflammatory factor and pain transmitter to mediate a variety of joint inflammatory conditions (Denk et al., 2017). NGF not only regulates the proliferation and differentiation of bone neoplasms, osteodynia, and chondrocytes through activation of its high-affinity receptor TrkA, which affects the PI3K, Ras, and PLC pathways, but also helps to slow the progression of arthritis (Figure 5). Therefore, nerve growth factor has received widespread attention as a potential therapeutic target for arthritis. Some researchers have found that blocking nerve growth factor signalling by inhibiting TrkA reduces pain in osteoarthritic rats (Nwosu et al., 2016). As a result, most studies now suggest that inhibition of nerve growth factor and TrkA reduces patient pain and delays disease progression. However, due to the pleiotropic nature of NGF action, the effect of inhibiting NGF-TrkA signalling in arthritis treatment remains controversial, and it is not clear whether there are significant negative effects (Walsh and Neogi, 2019). In patients with chronic arthritis, TrkA expression was significantly reduced in peripheral blood and synovial fluid mononuclear cells, resulting in a loss of inhibitory effect of nerve growth factor on inflammatory cytokine release, suggesting that inhibition of the nerve growth factor-TrkA signalling pathway may instead adversely affect patients with arthritis (Prencipe et al., 2014). There is also evidence that nerve growth factor receptors are upregulated in osteoblasts during arthritis and play an important role in remodelling and repair of osteoarthritic joints, while inflammatory factors (e.g., IL-1β, TNF-α) and mechanical stress stimuli have been found to promote NGF expression in articular cartilage and synovial tissue, further exacerbating pain (Zhao L. et al., 2024). Current research into osteoarthritis has found that inhibition of nerve growth factor signalling leads to impeded and delayed bone remodelling in osteoarthritic joints (Zhao L. et al., 2024). Schnitzer and Marks found that NGF inhibitors significantly relieved patients’ pain symptoms compared with placebo through a systematic analysis of 13 clinical trials in hip or knee OA. These findings provide an important theoretical basis for NGF-targeted treatment of arthritis (Schnitzer and Marks, 2015).

### 6.2 Osteoporosis

Osteoporosis (OP) is a systemic skeletal disease characterised by a decrease in bone mass and deterioration of bone microarchitecture, the prevalence of which increases significantly with the ageing of the population. The clinical manifestations of OP mainly include osteodynia, pathological fractures and activity dysfunction, and its pathogenesis is complex, involving an imbalance between reduced osteogenic function and increased osteoblastic activity (Wang et al., 2021). The potential of NGF in OP treatment is an emerging area of research. Studies have shown that bone marrow and serum levels of NGF are significantly elevated in patients with osteoporosis and negatively correlate with bone mineral density (BMD). The prevalence of OP increases with the ageing of the population and is associated with an increased risk of fracture and chronic pain, and the targeting of NGF with chelating antibodies ameliorates musculoskeletal pain including back pain and arthritis (Suzuki et al., 2018). Another study suggests that NGF may promote differentiation of MSCs to osteoblasts and enhance bone formation by activating the TrkA receptor signalling pathway (Chen et al., 2021). As a large molecular protein, systemic administration of NGF may face low bioavailability, and engineered exosomes or targeted delivery systems may be the solution. The direct role of NGF in osteoporosis treatment is unclear, and future studies should focus on how NGF affects bone metabolism through neuromodulation and explore its synergistic effects with existing anti-osteoporosis drugs.

### 6.3 Bone neoplasms

Current clinical data show that NGF in bone neoplasms treatment mainly focuses on the treatment of neurogenic tumours and peripheral nerve injury, but its mechanism of action and potential application in bone tumours should not be ignored. Some studies have pointed out that the expression of NGF in osteosarcoma tissues is significantly higher than that of other growth factors, and cellular experiments have demonstrated that NGF promotes the metastasis of osteosarcoma cells by up-regulating matrix metalloproteinase 2 expression (Hou et al., 2024). In addition, NGF has a promotional role in peripheral nerve regeneration, and the microenvironment of bone neoplasms is often accompanied by neural infiltration, which may affect tumour growth and metastasis. Nerve growth factor, as a neurogenic signalling factor, may play an important role in the metastasis of many malignant tumours to bone, including breast cancer, oral squamous cell carcinoma and prostate cancer (Shan et al., 2022; Zhang Y. et al., 2023). The association of nerve growth factor with bone neoplasms is related to its high-affinity receptor TrkA, and the gene encoding TrkA, NTRK1, may undergo fusion mutations, resulting in sustained activation of downstream pathways (Yichao et al., 2024). Meanwhile, NGF may affect tumour invasion by regulating the activity of tumour-associated nerves. Studies suggest that NGF may promote the progression of malignant tumours such as osteosarcoma by activating TrkA or p75 receptors (Zhou et al., 2016). On the other hand, NGF may also exert anti-tumour effects by inducing apoptosis or inhibiting angiogenesis in tumour cells (Ferraguti et al., 2024). Monoclonal antibody targeting NGF, used in clinical trials for cancer-related bone pain, shows some analgesic effect (Ma et al., 2022). NGF can promote the expression of VEGF and indirectly affect the blood supply of the tumour, thus influencing its growth and metastasis. Immune escape in the tumour microenvironment is one of the major challenges in the treatment of bone neoplasms. NGF may affect the immune microenvironment of the tumour by regulating the function of immune cells, such as macrophages and T cells. Currently, exosomes have been explored as drug delivery carriers, and NGF may be an adjunct to targeted therapy if it specifically binds to tumour-associated nerves or blood vessels.

### 6.4 Osteodynia

**FIGURE 6:**
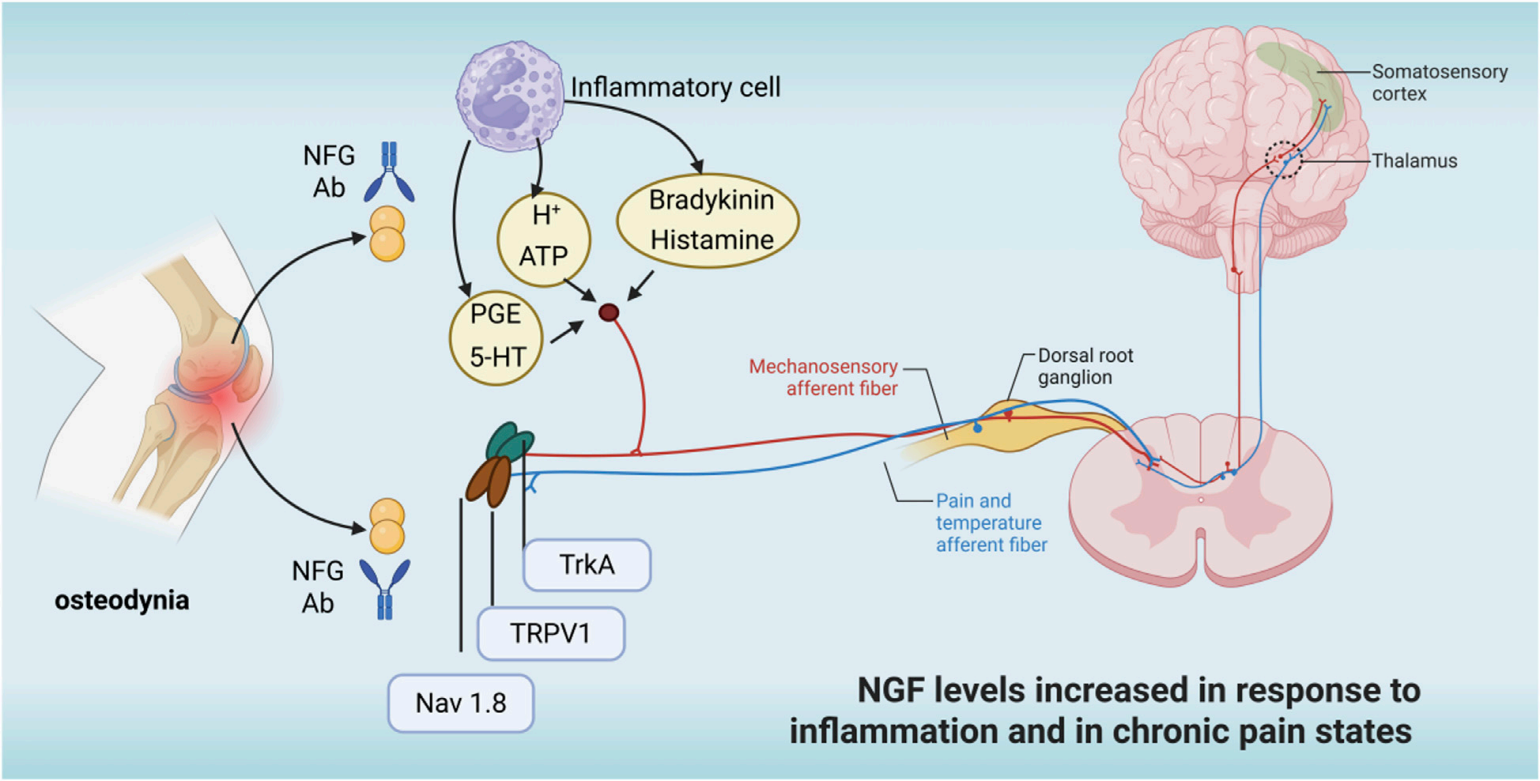
NFC increases in the osteodynia state.

As shown in Figure 6, nerve growth factor receptors are expressed in most bone injury receptors, and exogenous nerve growth factor can bind to TrkA receptors via NGF, activating downstream signalling pathways and increasing neuronal excitability, leading to nociception (Nencini et al., 2017). Long-term internalised NGF-TrkA complexes are retrogradely transported and the synthesis of substance P, calcitonin-generating peptide (CGRP), brain-derived neurotrophic factor (BDNF), sodium and calcium channels were increased. These effects lead to a lower threshold of activation of nociceptors and increased pain sensation. In conditions such as osteoarthritis or fractures, inflammatory cells and damaged bone tissue release large amounts of NGF, exacerbating pain. Sensory nerve fibres in bone tissue overgrow in pathological states and NGF promotes this abnormal innervation, which is associated with chronic pain. It has been shown that the acute behavioural response of NGF to bone can be at least partly mediated by the rapid activation of mechanically activated bone nociceptors, NGF chelation for osteogenic pain (Nencini et al., 2017). It has also been shown that anti-NGF therapy is efficacious in preventing pain-inducing adaptations in the functional brain network after sustained injurious inputs of cancer-induced osteodynia (Buehlmann et al., 2019). Yao et al. randomly grouped rats and then injected 10 μL of phosphate buffer or Walker256 tumour cells into the upper left tibia. Thirteen days after the injection, intrathecal catheter insertion was performed, followed by twice-daily injections of saline, anti-nerve growth factor, nerve growth factor and naloxone. Behavioural changes in pain were measured at set time points. The results showed that injection of tumour cells into the tibia resulted in nociceptive hypersensitivity and increased expression of μ-opioid receptor proteins and mRNAs in the dorsal horn of the spinal cord and dorsal root ganglia, compared with the sham-operated group. After intrathecal injection of anti-nerve growth factor, a significant anti-injury effect was seen with increased μ opioid receptor expression compared to the cancer pain group (Yao et al., 2016). In primary and secondary tumours of bone, tumour cells can induce sensory and sympathetic nerve endings to grow into the tumour tissue via the nerve growth factor-TrkA signalling pathway, leading to cancer-associated osteodynia (Yang et al., 2024; Yoneda et al., 2023). The degree of osteodynia increases progressively with tumour development and the underlying mechanisms are related to injury, inflammation, tumour invasion and a variety of other neuropathological processes (Jing et al., 2023; Yang et al., 2023). Researchers have also shown that blocking the osteodynia-TrkA signalling pathway by using specific neutralising antibodies can eliminate or reduce osteodynia without delaying bone healing (Rapp et al., 2015). However, Kan et al. (Kan et al., 2024) indicated that Nerve Growth Factor enhances analgesia in an experimental mouse model of cancer pain by up-regulating membrane-associated opioid receptors, suggesting that the Nerve Growth Factor-TrkA signalling pathway also plays an upstream regulatory role in the mechanism of analgesia.

### 6.5 Fracture

**FIGURE 7:**
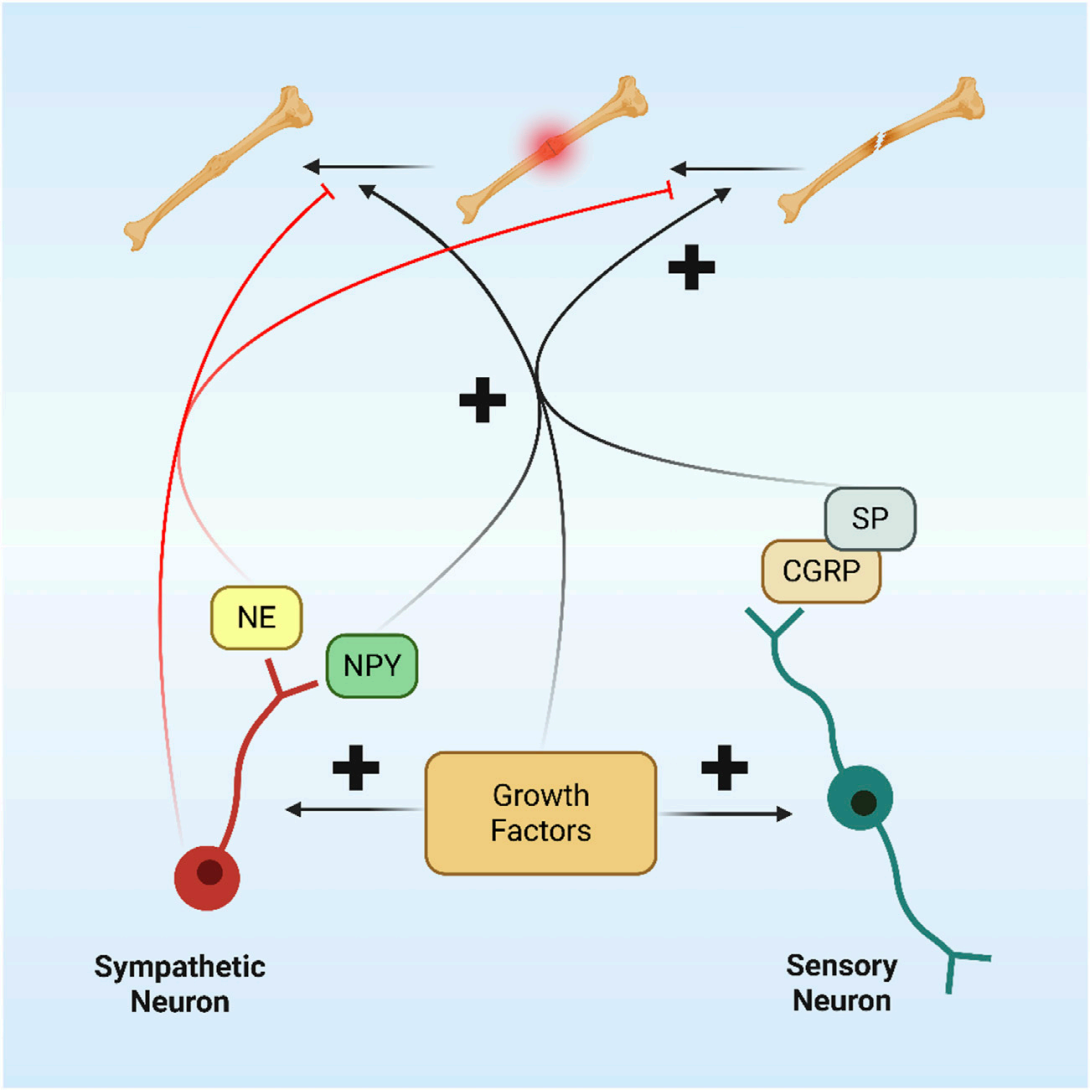
Nerve interaction in fracture healing.

In recent years, the potential application of NGF in fracture healing and bone tissue repair has also received increasing attention. Fracture healing is a complex biological process involving multiple stages such as inflammatory response, cartilage formation, and bone scab remodelling, and NGF may affect this process by regulating neuroskeletal interactions, promoting angiogenesis and osteoblast differentiation, etc (Figure 7). NGF intervention in tibial bone rats induced fracture healing. The mechanism of action may be related to the activation of the Wnt3α/β-catenin signalling pathway, which has a better effect of use (Zhao Y. et al., 2024). NGF upregulates the expression of fracture healing proteins to promote fracture healing. Yang et al. randomly divided 48 rabbits established in a fracture model of localised nerve injury to the mandible into a nerve growth factor group (NGF group), a gelatin sponge group (GS group), a blank group and an intact group. The pinprick response of the lower lip at weeks 2 and 4 postoperatively showed that the number of animals with neurological reflex recovery was significantly higher in the NGF group than in the GS and blank groups. Macroscopic observation, CBCT examination, and histological analysis showed that there were a large number of osteoblasts and some vascular endothelial cells around the small bones in the NGF group, and the amount of bone call formation and reconstruction was better than that of the GS group in the second postoperative week. qRT-PCR results showed that the expression levels of BMP-9 and VEGF in the four groups reached the highest values in the second week, and the expression levels of both in the NGF group were significantly higher than that of the GS group. Exogenous NGF can promote the healing of mandibular fractures. This work will provide a new basic and theoretical foundation for elucidating the mechanism of fracture healing, thereby promoting fracture healing and reducing the incapacitation rate of patients (Yang et al., 2021). Joint replacement or fracture often results in skeletal pain that needs to be adequately controlled, and application of anti-NGF before orthopaedic surgery or after a fracture can reduce skeletal pain behaviour by 40%–70% (Majuta et al., 2015). Currently, NGF is used in combination with biomaterials for the repair of large-segment bone defects, or NGF genes are delivered through viral or non-viral vectors for local sustained expression (Fitzpatrick et al., 2021; Wang et al., 2024; Wang et al., 2023).

### 6.6 Effects on cartilage tissue

Articular cartilage, as a special connective tissue covering the ends of long bones, plays a key role in cushioning mechanical stress and maintaining joint function. However, due to the lack of blood vessels and nerves, it is difficult for cartilage to repair itself once it is damaged, which seriously affects the patient’s motor function and quality of life (Chen et al., 2025). NGF shows aberrant expression in a variety of bone and joint diseases, suggesting that it may be involved in the disease process. Intra-articular anti-VEGF antibody reduces phosphorylated VEGFR2 levels in articular chondrocytes and synoviocytes and reduces phosphorylated VEGFR1 levels in dorsal root ganglia. NGF exacerbates joint inflammation by activating the VEGF signalling pathway, which promotes the activation of inflammatory cells and the release of inflammatory mediators. This process is particularly important in OA and is one of the key factors in the development of OA (Nagao et al., 2017). In addition, in studies based on a mouse fracture model, it was found that significant nerve growth factor expression occurred in cartilage healing tissues, whereas the enhancement of nerve growth factor expression by transgenic technology significantly promoted cartilage growth and differentiation and chondrocyte differentiation by expression of the SOX9 gene, a transcription factor. The effects of nerve growth factor on cartilage tissue are mainly indirect through the effects on nerves and blood vessels (Haseeb et al., 2021). Similarly, a dramatic increase in nerve growth factor expression in cartilage healing tissues early in the trauma process subsequently triggers neurovascular growth, and the use of TrkA agonists results in further maturation of vascular growth and the vascular network, with a significant enhancement of cartilage repair, which can even lead to heterotopic ossification. NGF provides the necessary microenvironment support for cartilage repair by promoting the growth of nerves and blood vessels. The growth of nerves and blood vessels not only provides nutrients for chondrocytes but also further promotes their survival and function by releasing neurotrophic factors and angiogenic factors (Cherief et al., 2022). It has been reported that *in vitro* angiogenic activity of endothelial cells was significantly enhanced after co-culture with chondrocytes pretreated with NGF, and knockdown of TrkA receptor on chondrocytes significantly eliminated the above effect. This study suggests that NGF may not only have direct effects on peripheral nerves and blood vessels, but may also affect vascular endothelial cells by activating the PI3K/Akt signalling pathway via chondrocytes (Yu et al., 2019).

## 7 NGF targeted therapy and challenges

NGF and its receptor play key roles in skeletal system, neurological diseases, cancer and angiogenesis, but its targeted therapy still faces many challenges. NGF plays an important role in nerve repair, but may also exacerbate inflammatory responses, e.g., in OA, NGF may promote pain signalling and cartilage degradation. NGF may cause central nervous system side effects such as ataxia, sensory abnormalities, and even accelerated side effects such as structural deterioration of joints. NGF may also cause side effects such as ataxia, sensory abnormalities, and even accelerated joint deterioration. NGF or its inhibitors need to be delivered precisely to the lesion site to avoid affecting normal tissues with insufficient targeting. NGF injections for diabetic neuropathy have been abandoned due to pain at the injection site, and alternative factors have been explored. Some NGF-targeted therapies can relieve pain but may affect neuroplasticity in the long term. NGF functions through TrkA and p75NTR, but the functions and signaling pathways of these two receptors vary in different cell types. How to achieve receptor selectivity and avoid non-specific activation or inhibition is an important task for future clinical treatment. Combining NGF with other growth factors synergistically promotes bone formation and angiogenesis to improve therapeutic efficacy. Development of biomarkers based on the NGF signalling pathway for predicting patient response to NGF therapy enhances the clinical role.

## 8 Conclusion

NGF plays an important role in skeletal physiology and pathology and is an important target for the treatment of skeletal diseases. NGF plays an important role in bone formation by binding to TrkA receptor and activating the PI3K-Akt and Ras-MAPK signalling pathways to promote the proliferation and differentiation of osteoblasts. At the same time, NGF affects the activity of osteoclasts and regulates bone resorption through the p75NTR-mediated signalling pathway. During bone repair, NGF supports the regeneration of bone tissue by promoting nerve regeneration and angiogenesis. The discovery of these mechanisms provides a theoretical basis for NGF-targeted therapy. In the future, the application of NGF in the treatment of skeletal diseases will be more promising with the deepening of related research and the expansion of clinical applications. By optimising target specificity, drug delivery systems and combination therapy strategies, the current challenges can be overcome and the widespread application of NGF therapy can be realised.
